# Setup margins based on the inter‐ and intrafractional setup error of left‐sided breast cancer radiotherapy using deep inspiration breath‐hold technique (DIBH) and surface guided radiotherapy (SGRT)

**DOI:** 10.1002/acm2.14271

**Published:** 2024-01-25

**Authors:** Volker Rudat, Yanyan Shi, Ruping Zhao, Wei Yu

**Affiliations:** ^1^ Department of Radiation Oncology Jiahui International Cancer Center Shanghai, Jiahui Health Shanghai China

**Keywords:** breast cancer radiotherapy, deep inspiration breath‐hold, DIBH, patient positioning, SGRT, surface guided radiation therapy

## Abstract

**Purpose:**

The use of volumetric modulated arc therapy (VMAT), simultaneous integrated boost (SIB), and hypofractionated regimen requires adequate patient setup accuracy to achieve an optimal outcome. The purpose of this study was to assess the setup accuracy of patients receiving left‐sided breast cancer radiotherapy using deep inspiration breath‐hold technique (DIBH) and surface guided radiotherapy (SGRT) and to calculate the corresponding setup margins.

**Methods:**

The patient setup accuracy between and within radiotherapy fractions was measured by comparing the 6DOF shifts made by the SGRT system AlignRT with the shifts made by kV‐CBCT. Three hundred and three radiotherapy fractions of 23 left‐sided breast cancer patients using DIBH and SGRT were used for the analysis. All patients received pre‐treatment DIBH training and visual feedback during DIBH. An analysis of variance (ANOVA) was used to test patient setup differences for statistical significance. The corresponding setup margins were calculated using the van Herk's formula.

**Results:**

The intrafractional patient setup accuracy was significantly better than the interfractional setup accuracy (*p* < 0.001). The setup margin for the combined inter‐ and intrafractional setup error was 4, 6, and 4 mm in the lateral, longitudinal, and vertical directions if based on SGRT alone. The intrafractional error contributed ≤1 mm to the calculated setup margins.

**Conclusion:**

With SGRT, excellent intrafractional and acceptable interfractional patient setup accuracy can be achieved for the radiotherapy of left‐sided breast cancer using DIBH and modern radiation techniques. This allows for reducing the frequency of kV‐CBCTs, thereby saving treatment time and radiation exposure.

## 1 INTRODUCTION

Deep inspiration breath‐hold (DIBH) is a commonly used technique in left‐sided breast cancer radiotherapy. Deep inspiration increases the distance from the chest wall to the heart. Compared to free breathing, deep inspiration during radiotherapy has been shown to reduce the dose to the heart by 26.2% to 75.0%.^1^ To achieve the planned dose distribution, it is important to keep the extent of the deep inspiration breath‐hold as well as the position of the left breast or chest wall constant between and within the radiotherapy fractions. When using advanced radiation techniques such as volumetric modulated arc therapy (VMAT), simultaneous integrated boost (SIB), and hypofractionation protocols, patient setup accuracy is increasingly important. The highly conformal dose distributions produced by VMAT require adequate patient setup accuracy to obtain optimal outcomes. Hypofractionation protocols may be more sensitive to patient setup errors compared to conventional fractionation protocols due to the significantly reduced number of radiotherapy fractions. Surface guided radiotherapy (SGRT) is an optical technology that allows monitoring the patient's surface with sub‐millimeter precision in real‐time.^2^ Due to the close proximity of the target volume breast or chest wall to the skin, the patient's surface is an excellent surrogate for accurate patient setup. While excellent intrafractional setup accuracy has in general been reported during DIBH using SGRT, greater deviations have also been observed.^3^, ^4^ Many workflow and patient‐related factors may impact the patient positioning accuracy; for example, the DIBH technique used (voluntary, spirometry, x‐ray, infrared markers),^5^ the gating protocol (threshold value, automatic, or manual gating), the use of a visual feedback system for the patient to monitor the correct thorax position during DIBH,^6^, ^7^ conducting a pre‐treatment training for DIBH,^8^ the patient compliance, the thorax shape,^9^, ^10^, ^11^ and other factors. The patients in our analysis were positioned for radiotherapy using daily SGRT and kilovolt cone‐beam computed tomography scans (kV‐CBCT). The interfractional patient setup accuracy was represented by the six degrees of freedom (6DOF) shifts based on kV‐CBCT. The intrafractional patient setup accuracy using DIBH was represented by the SGRT readings during beam application.

The goal of our study was to estimate the inter‐ and intrafractional patient setup accuracy using the advanced radiation techniques VMAT, SIB (where indicated), DIBH, SGRT, hypofractionation, pre‐radiotherapy training for DIBH and use of visual feedback system during DIBH for the radiotherapy of left‐sided breast cancer. Furthermore, setup margins to compensate for the patient setup error were calculated using the van Herks formula for the scenarios online verification using daily SGRT combined with kV‐CBCT every day, every second day, once a week, or no kV‐CBCT. The purpose of this analysis was to estimate if the frequency of kV‐CBCTs can be reduced to save treatment time and radiation exposure while keeping adequate patient setup accuracy.

## 2 PATIENTS AND METHODS

The inclusion criteria for this retrospective analysis consisted of breast cancer patients receiving adjuvant left‐sided radiotherapy using voluntary DIBH, daily SGRT, and daily kV‐CBCT after breast‐conserving surgery or mastectomy. Patients who could not hold their breath for 30 s were excluded from the analysis.

### 2.1 Optical surface scanning system

AlignRT (VisionRT, London, Great Britain) is an optical tracking system that uses three ceiling‐mounted stereo video camera pods to reconstruct the three‐dimensional (3D) surface of the patient. Each pod includes a red‐light projector and two camera sensors. Each camera pod projects a visible red light with a pseudo‐random speckle pattern onto the patient's body. The AlignRT software generates a real‐time 3D surface image of the patient. This surface is compared with a reference surface within the region of interest defined by the user to derive a 6DOF shift representing the patient's position in real‐time. For the reference surface, a reconstructed 3D surface derived from the simulation computed tomography (CT) image (Dicom surface) or a 3D surface captured by AlignRT can be used. The region of interest used in our study was the left breast for patients after breast conserving surgery, or the left chest wall for patients after mastectomy.

Based on the kV‐CBCT, the interfractional setup error with AlignRT was reflected by the shift of the Hexapod 6D treatment couch. During radiotherapy in DIBH, the difference between the AlignRT surface image and the reference surface image was a measure of the intrafractional setup error.

### 2.2 Preparation for DIBH

At the first consultation, patients received an appointment for the introduction and practice of the voluntary DIBH technique using visual feedback at the CT‐simulator. The patients were asked to hold their breath in deep inspiration for 30 s using visual guidance as feedback on the breath hold position. In addition, the patients were given written instructions on how to practice DIBH at home. Routine practice was that patients who are not able to reliably hold their breath for 30 s after practicing were treated with free breathing (FB).

### 2.3 CT‐simulation

Patients were simulated in the supine position. A breast board (Civco Medical Instruments Co Inc., Orange, IA, USA) and a knee fix were utilized as positioning devices. CT‐simulation was accomplished using a Brilliance Big Bore CT (Philips, Amsterdam, the Netherlands). The slice thickness was 3 mm. Respiratory Gating for Scanners (RGSC, Varian Medical Systems, Palo Alto, CA, USA) was used to monitor the breathing motion.

### 2.4 Treatment planning

Target volumes (whole breast or chest wall, with or without locoregional lymph nodes) were contoured according to the RTOG Breast Cancer Atlas.^12^ Automatic contouring of organs at risk (OAR) was performed using AccuContour Version 3.1 (Manteia Technology LTD, Xiamen, China).

Monaco Version 5.40.03 was the treatment planning system (Elekta AB, Stockholm, Sweden). Mobius 3D Version 2.1 (Varian Medical Systems, Palo Alto, CA, USA) was utilized for the independent dose verification. DoseLab Version 6.8 (Varian Medical Systems, Palo Alto, CA, USA), ArcCheck Version 8.0.0.11708 (Sun Nuclear Corporation, Melbourne, FL, USA), and MatriXX (Iba Dosimetry GmbH, Schwarzenegger, Germany) were utilized for quality assurance.

Patients were usually treated with a hypofractionated radiotherapy regimen. The hypofractionated radiotherapy regimen consisted of 40.05 Gy in 15 fractions if no boost was prescribed, 40.0 Gy to the whole breast, and 48.0 Gy to the tumor bed in 16 fractions if a simultaneous integrated boost (SIB) was prescribed. Alternatively, patients could be treated with conventional fractionation (50.4 Gy in 28 fractions with or without a sequential boost of 10.0 Gy in five fractions).

Volumetric Modulated Arc Therapy (VMAT) was used for treatment planning. To reduce the beam‐on time, a 6 MV flattening filter free (FFF) beam was used. The dose rate was 1400 MU/min. Usually, two 60° partial arcs were delivered with 300°/360° and 105°/165° as start/stop angles, respectively. The delivery time of each arc was less than 30 s. For most patients, the arc could be delivered within one breath hold. For the treatment of locoregional lymph nodes, usually the arc length and, subsequently, the delivery time were increased. For these plans, some patients needed two breath holds for the delivery of an arc.

### 2.5 Treatment

Radiotherapy was delivered using VersaHD (Elekta AB, Stockholm, Sweden) with Agility MLC (5 mm leaves) and a Hexapod 6D treatment couch. Daily online verification was performed using kV‐CBCT, and translational and rotational errors were corrected by adjusting the Hexapod 6D treatment couch. In addition, the patient setup was verified using orthogonal kV/kV pair imaging with an electronic portal imaging device (EPID).

### 2.6 Clinical workflow

The workflow for the first radiotherapy fraction was demonstrated in Figure 1a, and for the subsequent fractions in Figure 1b. During the first radiotherapy fraction, the patient was first positioned using laser alignment with skin marks. This position was adjusted using AlignRT with the free breathing (FB) Dicom surface as a reference. The patient was then coached into the DIBH position using visual feedback, and the DIBH position was further adjusted using AlignRT with the DIBH Dicom surface as a reference. After which, a kV‐CBCT in FB was acquired, and a couch shift was performed based on the image registration with an FB simulation CT. An AlignRT reference surface was obtained in FB. A kV‐CBCT was then performed in DIBH, and a couch shift was performed based on the image registration with the DIBH simulation CT scan. A new AlignRT reference surface was obtained, and an orthogonal kV/kV pair image in DIBH was taken for final verification. If the patient setup was not within the limits of 3 mm, the patient setup was corrected, and this step was repeated. If the patient setup was within the limits, the radiotherapy treatment proceeded (Figure 1a). The kV‐CBCT in FB at the first fraction was performed because it helped to improve the patient setup accuracy. The alignment with the spine was more accurate with kV‐CBCT in FB, allowing a more precise monitoring of the breathing amplitude.

FIGURE 1(a) Workflow of the first radiotherapy fraction. (b) Workflow of the radiotherapy fractions after the first.

**Figure:**
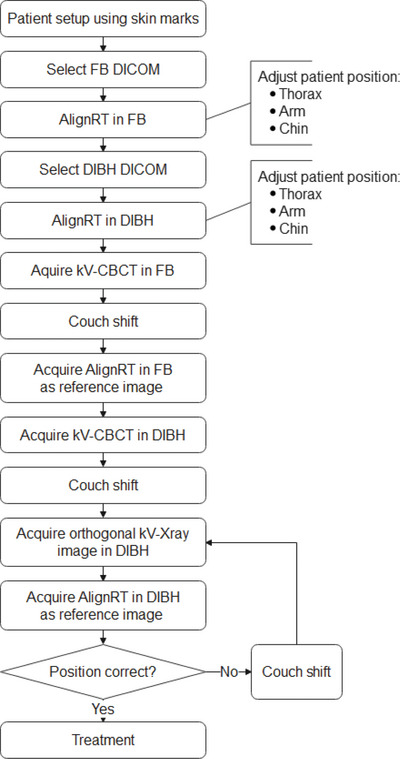

**Figure:**
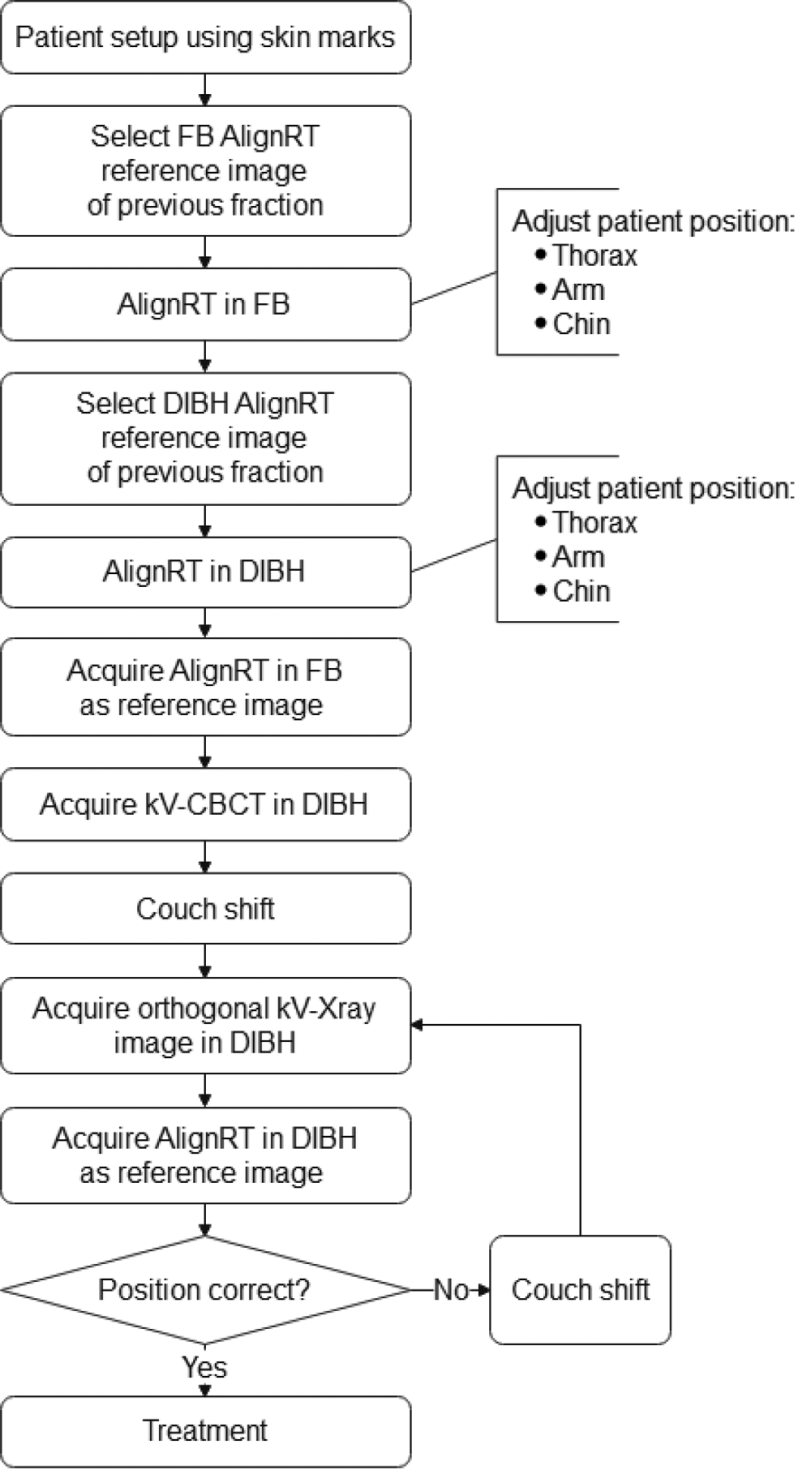

The workflow for subsequent fractions was shorter. The main difference was that the patient setup was adjusted using the AlignRT reference image in FB and DIBH obtained during the previous fraction. The AlignRT reference image of the previous fraction was chosen as a reference because it was considered to probably be more accurate for the measurement of the setup accuracy compared to the AlignRT reference image of the first radiotherapy fraction. The time for a radiotherapy fraction from patient setup to treatment finish was, on average, 15 min.

### 2.7 Data collection

**FIGURE 2:**
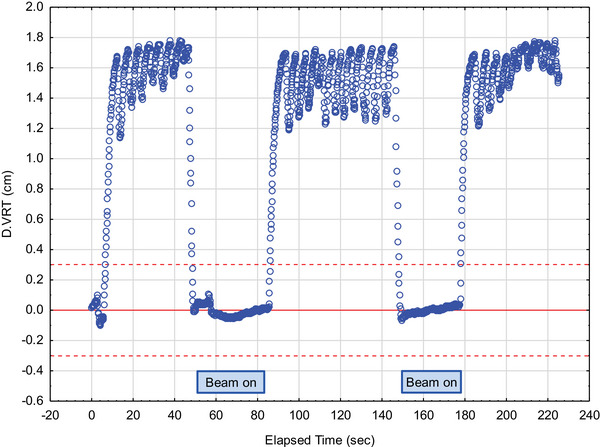
Time plot showing the vertical breathing curve amplitude over time. The dashed red lines represent the lower and upper limit of the gating window, and the solid red line the reference position.

The hospital information system (HIS) and the integrated oncology management system MOSAIQ (Elekta AB, Stockholm, Sweden) provided patient and treatment‐related data. The data were imported into a custom‐built database (Access, Microsoft, Redmont, USA). The anonymized data were then transmitted to a statistical software program for statistical analysis (Statistica, TIBCO Software Inc., 2020. Data Science Workbench, version 14. http://www.statsoft.com). The AlignRT RealTimeDelta text files were transferred into the statistical software program Statistica for analysis. Figure 2 shows an example of the data exported from AlignRT.

### 2.8 Statistical analysis

The patient setup accuracy was assessed by calculating the overall population mean setup error (M), the population systematic (Σ), and population random error (σ) of the translational and rotational errors in three directions (lateral, longitudinal, and vertical). The calculations were performed according to the report “On target: ensuring geometry accuracy in radiotherapy” by the Royal College of Radiologists.^13^ Accordingly, the overall population mean setup error (*M*) was defined as the overall mean of the analyzed patient group, the population systematic error (Σ) as the standard deviation of the individual mean set‐up error about the overall mean (*M*), and the population random error (*σ*) as the mean of all individual random errors.

Treatment margins to compensate for the patient setup error were estimated using the van Herk formula.^14^ Accordingly, the margin required to ensure 95% minimum dose to the planning target volume (PTV) for 90% of the patients was given by

(1) $$M_{\mathrm{PTV}}=\;2.50\;\sum 1.64\;\sigma -1.64{\;\sigma }_{P}$$

where Σ is the square root of the quadratic sum of the standard deviations of all contributing systematic errors, σ the square root of the quadratic sum of the standard deviations of all contributing random errors, and *σ*_P_ the standard deviation describing the width of the penumbra. The representative standard deviation of the penumbra width *σ*_P_ of the linear accelerator was 3.2 mm.

To simulate the impact of the frequency of online verifications using kV‐CBCT every day, every other day, once per week, or no kV‐CBCT on the calculated setup margin, the patient set‐up parameters were calculated assuming a patient setup error of 0 mm in all directions after online verification using kV‐CBCT.

To test differences in the patient setup accuracy for statistical significance an analysis of variance (ANOVA) was performed. The dependent variable was the individual standard deviation of the patient setup errors, and the independent variables were the direction of the patient setup error (lateral, longitudinal, and vertical) and the type of patient setup error (interfractional vs. intrafractional).

## 3 RESULTS

Twenty‐three consecutive unselected breast cancer patients receiving left‐sided adjuvant radiotherapy of the breast or chest wall using DIBH, daily SGRT, and daily kV‐CBCT were analyzed in this study. All patients received DIBH pre‐treatment training and visual feedback during DIBH. During the study period, one of 24 left‐sided breast cancer patients could not hold her breath for 30 s and was excluded from the analysis. Twenty‐one of 23 eligible patients were treated using moderate hypofractionation. All patients were treated using VMAT. Fourteen patients received a simultaneous integrated boost (SIB). The locoregional lymph nodes were included in the planning target volume of eight of 23 patients (Table 1).

**TABLE 1:** Patient and treatment characteristics.

Characteristic		*N*	%
Age			
	Mean (SD)	45 (10) years	
	Minimum	28	
	Maximum	66	
T classification			
	ypT0	2	8.7
	pTis	1	4.3
	pT1	10	43.5
	ypT1	2	8.7
	pT2	5	21.7
	ypT2	2	8.7
	ypT3	1	4.3
N classification			
	N0	14	60.9
	N1	5	21.7
	N2	3	13.0
	N3	1	4.3
Fractionation regimen			
	Hypofractionation	21	91.3
	Conventional	2	8.7
Target volume			
	WBRT	16	69.6
	CW	7	30.4
Boost			
	SIB	14	60.9
	SEB	2	8.7
	No boost	7	30.4
Locoregional lymph nodes			
	No	15	65.2
	Yes	8	34.8

Abbreviations: CV, chest wall; SEB, sequential boost; SIB, simultaneous integrated boost; WBRT, whole breast irradiation.

**FIGURE 3:**
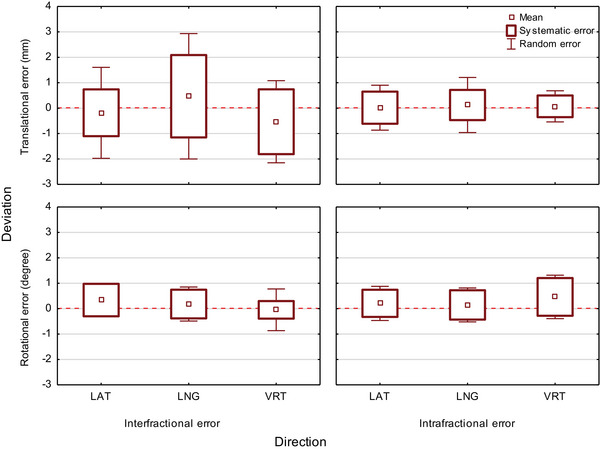
Box plot showing the inter‐ and intrafractional translational and rotational mean setup error, systematic error, and random error in the lateral, longitudinal, and vertical directions.

Three hundred and three radiotherapy fractions of 23 left‐sided breast cancer patients using DIBH and SGRT were used for the analysis. Figure 3 demonstrates that the translational intrafractional systematic and random setup errors were considerably smaller than the corresponding interfractional setup errors. An automated beam‐hold function of AlignRT stopped the beam at intrafractional deviations greater than 3 mm. The corresponding graphs represent the intrafractional variability within this limit. Figure 3 also shows that the systematic and random translational interfractional setup errors were greater in the longitudinal direction than in the lateral or vertical direction. The ANOVA showed that the differences in the mean standard deviation of the setup error were statistically significant in both the direction (longitudinal, 1.7 mm; lateral, 1.3 mm; vertical, 1.1 mm; *p* < 0001) and the type of the patient setup error (interfractional, 1.9 mm; intrafractional, 0.8 mm; *p* < 0.001).

An explanation for the lower interfractional setup accuracy in the longitudinal direction may be that the flat shape of the chest wall with limited curvature in the longitudinal direction is challenging for accurate SGRT localization. Other possible contributing factors could be the remaining variability of the left arm position despite correction or a varying degree of stretching of the spine in the supine position. The intrafractional systematic and random errors were considerably smaller in the vertical direction compared to the lateral and longitudinal directions. This observation suggests that the patient position in the vertical direction is the most robust during left‐sided breast radiotherapy using DIBH and AlignRT.

The inter‐ and intrafractional rotational errors were similar in all directions (<1°).

Table 2 presents the calculated setup margins in dependence on the frequency of online verifications using kV‐CBCT. As expected, due to the slightly lower patient setup accuracy using AlignRT compared to kV‐CBCT, the calculated interfractional setup margins became smaller with increasing frequency of online verifications using kV‐CBCT. The setup margin for the intrafractional error using AlignRT with an automated beam‐off threshold of deviations greater than 3 mm was, on average, ≤2 mm. The intrafractional setup error contributed ≤1 mm to the combined inter‐ and intrafractional setup margin. Depending on the frequency of online verifications using kV‐CBCT, the combined inter‐ and intrafractional setup margin ranged from maximal setup correction with daily kV‐CBCT to 4, 6, and 4 mm in the lateral, longitudinal, and vertical direction with SGRT alone.

**TABLE 2:** Inter‐ and intrafractional errors, and calculated setup margins in dependence on the frequency of online verifications.

			Interfractional error			Intrafractional error			Setup margin^a^		
Direction		Frequency of online verifications using kV–CBCT	*M*	Σ	*σ*	*M*	Σ	*σ*	Inter‐fractional error	Intra‐fractional error	Combined inter‐ and intra‐fractional error
Translational error (mm)											
	LAT	None	−0.2	0.9	1.8	0.0	0.6	0.9	3	2	4
	LNG	None	0.4	1.6	2.4	0.1	0.6	1.0	5	2	6
	VRT	None	−0.6	1.3	1.6	0.1	0.4	0.6	4	1	4
	LAT	1/week	0.0	0.8	1.7	0.0	0.6	0.9	3	2	3
	LNG	1/week	0.2	1.5	2.2	0.1	0.6	1.0	5	2	5
	VRT	1/week	−0.6	1.0	1.5	0.1	0.4	0.6	3	1	3
	LAT	Every other day	0.0	0.5	1.4	0.0	0.6	0.9	2	2	3
	LNG	Every other day	0.3	1.0	1.9	0.1	0.6	1.0	3	2	4
	VRT	Every other day	−0.3	0.6	1.3	0.1	0.4	0.6	2	1	2
Rotational error (°)											
	LAT	None	0.3	0.6	0.7	0.2	0.7	0.5	–	–	–
	LNG	None	0.2	0.7	0.6	0.2	0.7	0.6	–	–	–
	VRT	None	−0.1	0.8	0.4	0.4	0.8	0.7	–	–	–
	LAT	1/week	0.2	0.6	0.5	0.2	0.7	0.5	–	–	–
	LNG	1/week	0.1	0.7	0.4	0.2	0.7	0.6	–	–	–
	VRT	1/week	0.0	0.7	0.3	0.4	0.8	0.7	–	–	–
	LAT	Every other day	0.1	0.5	0.3	0.2	0.7	0.5	–	–	–
	LNG	Every other day	0.1	0.5	0.3	0.2	0.7	0.6	–	–	–
	VRT	Every other day	0.0	0.6	0.2	0.4	0.8	0.7	–	–	–

Abbreviations: kV‐CBCT, Kilovoltage cone beam computed tomography; LAT, lateral; LNG, longitudinal; M, overall population mean set‐up error; VRT, vertical; Σ, population systematic error; σ, population random error.

^a^ Calculated setup margins according to the van Herk formula.

## 4 DISCUSSION

Our data show that with SGRT an excellent intrafractional and acceptable interfractional patient setup accuracy can be achieved for the radiotherapy of left‐sided breast cancer using modern techniques. This allows reducing the frequency of kV‐CBCTs thereby saving treatment time and radiation exposure.

The use of advanced radiation techniques (e.g., VMAT, SIB) and hypofractionated protocols require adequate patient setup accuracy to obtain optimal results. A margin to compensate for the setup error of 4, 6, and 4 mm in the three directions using SGRT without kV‐CBCT during radiotherapy appears to be acceptable. However, it should be noted that only the geometric patient setup accuracy was analyzed in our study. Other factors, like changes in the shape of the breast during radiotherapy or the dose distribution itself, were not considered or analyzed. Furthermore, pre‐radiotherapy DIBH training for the patient that includes the use of a visual feedback system for the patient to monitor the correct thorax position during DIBH is required to achieve these relatively small margins. This may be considered a disadvantage because it costs additional time.

We observed that the interfractional patient setup error was significantly greater in the longitudinal compared to the lateral or vertical direction. Future research should be directed to reduce this error, for example, by optimizing the region of interest (ROI) or applying additional hardware like transponders.

Our results are well in line with the results of other study groups. In an analysis of 6013 deep inspiration breath‐holds of 103 left‐sided breast cancer patients, the median standard deviation of the breath‐hold level during DIBH was 0.3 mm, and the maximum difference in the breathing amplitudes on average 1.3 mm.^15^ However, only the vertical amplitude could be evaluated with the Catalyst system employed in this study. An analysis of 1705 breath‐holds in 261 fractions of 18 patients with left‐sided breast cancer using DIBH, AlignRT, and an air‐volume guidance system (ABC, R3.0, Elekta AB, Stockholm, Sweden)^10^ showed an intrafractional deviation of −0.7 mm (2.9 mm) on average (standard deviation). An intra‐DIBH stability of equal or less than 0.7 mm and intrafractional reproducibility of equal or less than 2.2 mm were found in an analysis of 1305 fractions of 58 breast cancer patients receiving left‐ or right‐sided SGRT using AlignRT.^5^ In a study of 228 people with left‐sided breast cancer who were treated with DIBH and AlignRT, the average reproducibility was 1.69 and 1.30 mm in the subgroup of 10 people who got visual feedback during the DIBH.^7^ An analysis of 40 left‐sided breast cancer patients treated using DIBH and AlignRT revealed a median intrafractional deviation of approximately 1 mm in all directions.^3^ A subgroup of 27 of the 67 left‐sided breast cancer patients who had DIBH training before their CT‐simulation were found to have maximum intrafractional chest wall excursions with an average (SD) of 2.5 mm (0.6 mm).^3^ The chest wall excursions were measured using real‐time surface tracking transponders (Calypso, Varian Medical Systems, Palo Alto, CA, USA). Delombaerde et al. looked at the intrafractional setup error of spirometer‐guided breath‐hold breast radiotherapy (SDX system, Dyn'R, France) using inbore surface monitoring and portal imaging on Halcyon (Varian Medical Systems).^11^ All patients received a coaching session before the CT‐simulation. The intrafractional systematic and random error in the longitudinal direction was 1.1 and 1.0 mm, and in the vertical direction 0.7 and 0.8 mm (our study: 0.6, 1.0 mm, and 0.4, 0.6 mm). The data were derived from 130 fractions of seven patients. Deviations in the lateral direction were not evaluated in this study. Intrafractional setup errors in a similar range have been reported in breast cancer patients receiving left breast cancer radiotherapy in free breathing. Using free breathing, intrafractional setup deviations in all axes with a standard deviation between 0.22 and 0.25 mm were observed in a study of 1170 fractions of 252 patients,^16^ between 1.06 and 1.53 mm in an analysis of 2028 fractions of 104 patients,^17^ and between 2.2 and 2.8 mm in an analysis of 292 fractions of 40 patients.^18^

The interfractional setup errors were significantly greater than the intrafractional setup errors in our study. Similar results were observed in a subgroup of 25 of 50 left‐sided breast cancer patients who were treated using DIBH and AlignRT.^9^ The interfractional systematic error was 1.1, 1.8, and 0.6 mm in the lateral, longitudinal, and vertical directions, and the random error 1.9, 2.9, and 2.0 mm, respectively. The calculated setup margin for the interfractional error was 3, 5, and 3 mm in the lateral, longitudinal, and vertical directions. Online correction using MV‐CBCT reduced the setup margins by approximately 1 mm. Intrafractional setup errors were not investigated in this study. A similar analysis of 1305 fractions of 58 left‐sided breast cancer patients treated using DIBH and AlignRT revealed an interfractional systematic error of 1.9, 1.3, and 1.5 mm in the lateral, longitudinal, and vertical directions and a random error of 2.2, 2.0, and 1.7 mm, respectively.^5^ The calculated setup margins were 6, 5, and 5 mm in the lateral, longitudinal, and vertical directions. Interfractional systematic and random errors below 2 mm in all directions were reported in an analysis of 143 fractions of 18 left‐sided breast cancer patients treated using DIBH and AlignRT,^10^ below 3 mm in all directions in an analysis of 47 patients,^7^ and 2.1 and 1.9 mm in the longitudinal and 0.8 and 1.2 mm in the vertical direction in an analysis of 140 spirometer‐guided breath‐hold breast treatments of seven patients using intra‐bore surface monitoring and portal imaging on Halcyon (Varian Medical Systems).^11^ Several studies, including ours^9^, ^11^, ^18^, ^19^ found the largest interfractional deviation using SGRT in the longitudinal direction. A possible explanation for this observation could be that the flat shape of the chest wall with limited curvature in the longitudinal direction is challenging for accurate SGRT localization. However, the largest interfractional deviation was not found in the longitudinal direction by several other study groups.^5^, ^10^, ^20^

There is general agreement that the interfractional setup accuracy using SGRT alone for left‐sided breast cancer patients is slightly inferior compared to using SGRT and IGRT with daily CBCT. Furthermore, there is general agreement that the interfractional setup accuracy using SGRT is slightly superior or at least equivalent compared to using laser alignment with skin marks. An analysis of 195 fractions from 76 breast cancer patients using laser alignment with skin marks instead of Catalyst^20^ showed that the median vector offset went down from 4.2 to 2.4 mm. In line with that, Penninkhof et al. reported a reduction of the population mean setup error from 3.9 to 1.4 mm using AlignRT,^7^ and Hattel et al. a reduction of the setup root‐mean‐square error from 5.4 to 4.2 mm using AlignRT.^21^ González‑Sanchis et al. found that breast surface matching was significantly improved from 92.7% to 98.0% in an analysis of 1170 fractions of 252 patients using laser alignment with skin marks compared to AlignRT.^16^ A reduction of the intrafractional setup error (standard deviation) using laser alignment with skin marks compared to Catalyst in the lateral, longitudinal, and vertical directions from 2.9 to 1.3 mm, 3.3 to 2.9 mm, and 2.9 to 1.4 mm was reported by Cravo Sa et al.,^19^ and from 6.1 to 2.4 mm, 3.8 to 2.7 mm, and 4.9 to 2.4 mm using Catalyst by Crop et al.^18^

## 5 CONCLUSIONS

An excellent intrafractional and acceptable interfractional patient setup accuracy can be achieved with SGRT for the radiotherapy of left‐sided breast cancer using DIBH and modern radiation techniques. This allows for reducing the frequency of kV‐CBCTs, thereby saving treatment time and radiation exposure.

## AUTHOR CONTRIBUTIONS

Volker Rudat designed the study, acquired, and analyzed the data, and drafted and finalized the manuscript. Yanyan Shi, Ruping Zhao, and Wei Yu contributed to the data acquisition, analysis, and interpretation of the data. All authors read and approved the final manuscript.

## CONFLICT OF INTEREST STATEMENT

The authors declare that they have no conflicts of interest.

## Data Availability

The data that support the findings of this study are available from the corresponding author upon reasonable request.
